# Update on Contrast-Induced Nephropathy: Recent Developments in Its Prevention, Early Diagnosis, and Therapy

**DOI:** 10.3390/medicina62050948

**Published:** 2026-05-13

**Authors:** Nazareno Carullo, Loredana Tripodi, Ashour Michael, Teresa Faga, Davide Bolignano, Giuseppe Coppolino, Yuri Battaglia, Nicola Ielapi, Davide Costa, Raffaele Serra, Michele Andreucci

**Affiliations:** 1 Renal Unit, “Magna Graecia” University of Catanzaro, I-88100 Catanzaro, Italy; 2 Department of Medical and Surgical Sciences, “Magna Graecia” University of Catanzaro, I-88100 Catanzaro, Italy; 3 Department of Health Sciences, “Magna Graecia” University, I-88100 Catanzaro, Italy; dbolignano@unicz.it (D.B.); davide.costa@unicz.it (D.C.); rserra@unicz.it (R.S.); 4 Nephrology and Dialysis Unit, San Giovanni di Dio Hospital, I-88900 Crotone, Italy; 5 Department of Medicine, University of Verona, I-37134 Verona, Italy; yuri.battaglia@univr.it; 6 Nephrology and Dialysis Unit, Pederzoli Hospital, Peschiera del Garda, I-37019 Verona, Italy; 7 Interuniversity Center of Phlebolymphology (CIFL), International Research and Educational Program in Clinical and Experimental Biotechnology, “Magna Graecia” University of Catanzaro, I-88100 Catanzaro, Italy

**Keywords:** renal failure, kidney, radiocontrast media, renal injury, cell death, cell injury, cell signalling, oxidative stress, glomerular filtration rate, acute kidney injury

## Abstract

Contrast-induced nephropathy (CIN), now more accurately referred to as contrast-induced acute kidney injury (CI-AKI), remains a major cause of hospital-acquired acute kidney injury (AKI) and is associated with increased morbidity and mortality, particularly in high-risk patients. This condition occurs following the intravascular administration of iodinated radiocontrast media (RCM), especially in individuals with pre-existing chronic kidney disease (CKD), diabetes mellitus, heart failure, advanced age, or exposure to high contrast volumes. The pathophysiology of CI-AKI is multifactorial and involves renal hemodynamic alterations, direct tubular toxicity, oxidative stress, inflammatory activation, and endothelial dysfunction, ultimately leading to tubular injury and reduced glomerular filtration rate (GFR). Traditional diagnostic markers such as serum creatinine (sCr) and estimated glomerular filtration rate (eGFR) are limited by low sensitivity and delayed response, prompting growing interest in novel biomarkers, including cystatin C (CysC), β-2 microglobulin (β-2M), Interleukin-18 (IL-18), Kidney Injury Molecule-1 (KIM-1), Neutrophil Gelatinase-Associated Lipocalin (NGAL), and osteopontin (OPN), which allow earlier detection and risk stratification. Preventive strategies remain the cornerstone of management and include optimizing hydration protocols, minimizing contrast dose, selecting low- or iso-osmolar agents, and individualized risk assessments. Despite extensive research into pharmacological and procedural interventions, no effective treatment for established CI-AKI exists, underscoring the critical importance of prevention and ongoing investigation into safer contrast agents and innovative prophylactic approaches.

## 1. Introduction

Contrast-induced acute kidney injury (CI-AKI) [previously known as contrast-induced nephropathy (CIN)] remains the third leading cause of hospital-acquired acute kidney injury (AKI) and continues to be linked with elevated morbidity and mortality rates, particularly among high-risk populations. CI-AKI is a well-recognized complication arising after the intravascular administration of iodinated radiocontrast media (RCM). It represents a significant cause of hospital-acquired AKI, particularly among patients with pre-existing renal impairment, diabetes mellitus, heart failure, or those undergoing complex interventional procedures, such as coronary angiography or computed tomography (CT) [1,2,3]. Iodinated RCM may be divided into different classes depending on whether they are ionic or non-ionic, whether they are monomeric or dimeric, and whether they are high-, low-, or iso-osmolar. A summary of some of the different types of iodinated RCM is shown in Table 1. In this article we summarize what is known regarding its prevention, early diagnosis, and therapy, incorporating the most recent findings reported in the scientific literature.

## 2. Definition

AKI following the administration of iodinated RCM does not necessarily imply a direct causal link to the contrast agent itself, which is why the terminology has now been further updated [4]: contrast-associated AKI (CA-AKI) or post-contrast AKI (PC-AKI) are both generic terms referring to AKI that occurs shortly after the administration of iodinated RCM and may or may not be directly caused by the RCM itself; and CI-AKI identifies more precisely the subgroup of PC-AKI in which a direct causal relationship to RCM administration is suspected (although a causal link cannot be established with certainty in epidemiological studies).

The causal relationship between RCM and AKI remains a matter of ongoing debate [5]. In recent years, increasing evidence has questioned whether all cases traditionally labeled as CI-AKI truly reflect direct nephrotoxicity, or rather represent coincidental or multifactorial AKI occurring in a high-risk clinical setting. This has led to the broader adoption of the terms CA-AKI and PC-AKI, which more accurately acknowledge the uncertainty of causality [2]. Notably, clinical observations show that many high-risk patients do not develop AKI following contrast exposure [6,7], whereas occasional cases occur even in individuals with apparently low risk profiles. This variability suggests that individual susceptibility, unmeasured confounders, and procedural factors play a significant role beyond contrast exposure alone. Furthermore, particularly in the setting of intra-arterial RCM administration, alternative mechanisms of renal injury must be considered. Cholesterol crystal embolization, often clinically silent and lacking peripheral signs, may account for a proportion of cases attributed to CI-AKI [8]. However, definitive diagnosis would require renal biopsy, which is rarely performed in this context, making it difficult to establish the exact etiology of AKI in many cases.

Therefore, while a direct nephrotoxic effect of contrast media is supported by experimental and clinical data, the true incidence of CI-AKI may be overestimated in observational studies. A careful interpretation of available evidence is warranted, and future studies should aim to better distinguish causal relationships from associative findings.

Following large-scale studies, several authorities in the fields of radiology and nephrology have adopted alternative terms (“PC-AKI” and “CA-AKI”) to refer to any AKI that occurs shortly after the administration of iodinated RCM [9]. The term CIN is also widely used in the literature and in clinical practice.

CI-AKI is traditionally defined as an increase in serum creatinine (sCr) of ≥0.5 mg/dL or ≥25% from baseline within 48–72 h after RCM exposure, in the absence of alternative causes. Ruling out other causes of AKI requires routine assessment with a thorough review of medical history, physical examination, urinalysis, and other relevant laboratory tests; moreover, renal ultrasound and renal biopsy may also be necessary.

Although the incidence varies depending on the patient population and any existing comorbidities, CI-AKI is estimated to occur in 2–7% of general patients and up to 50% in high-risk groups, such as those with pre-existing chronic kidney disease (CKD) or diabetes mellitus [10,11,12].

## 3. Pathophysiology of CI-AKI

**Figure 1:**
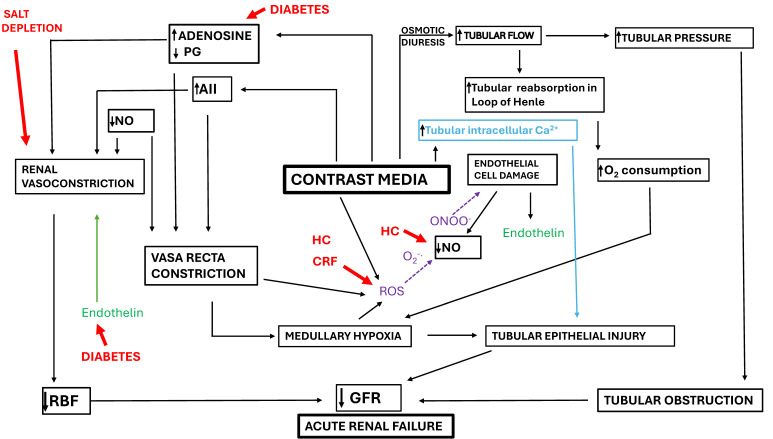
**The complex mechanisms by which RCM cause CI-AKI** [13]. Various clinical conditions, such as salt depletion, CKD, diabetes mellitus and hypercholesterolemia (HC) may aggravate the pathogenetic factors responsible for CI-AKI. PG: prostaglandin; CRF: Chronic Renal Failure; NO: Nitric Oxide; ROS: Reactive Oxygen Species; RBF: Renal Blood Flow; GFR: Glomerular Filtration Rate. The arrows in the text boxes indicate an increase (↑) or a decrease (↓).

The pathophysiology of CI-AKI is multifactorial and incompletely understood [13]. It involves a complex interplay of hemodynamic alterations, direct tubular cytotoxicity, oxidative stress, and inflammatory responses that ultimately lead to acute renal injury (Figure 1) [13]. These processes are particularly deleterious in patients with pre-existing renal impairment or comorbidities that compromise renal perfusion and oxygenation [14,15,16,17,18].

### 3.1. Renal Hemodynamic Alterations

Following intravascular administration of iodinated RCM, there is an initial transient renal vasodilation followed by a more prolonged vasoconstrictive phase, primarily mediated by adenosine, endothelin, and the activation of the renin–angiotensin–aldosterone system (RAAS) [17,19]. This vasoconstriction predominantly affects the renal outer medulla, an area already characterized by low oxygen tension, leading to medullary hypoxia. Simultaneously, a reduction in nitric oxide (NO) and prostaglandin availability contributes to impaired vasodilation, further exacerbating ischemic injury [20].

### 3.2. Direct Tubular Toxicity

Iodinated RCM exerts direct cytotoxic effects on renal tubular epithelial cells, with a particular predilection for the proximal tubules. These agents alter mitochondrial function, induce apoptosis and necrosis, and disrupt the cytoskeletal architecture [21,22]. Several intracellular signaling pathways, involving several proteins/kinases such as mitogen-activated protein kinase (MAPKs), protein kinase B (Akt/PKB), forkhead box O (FoxO) proteins, have been investigated to better understand the mechanisms of these toxic effects [23,24,25,26].

The high osmolarity and viscosity of RCM also increase tubular workload and intratubular pressure, aggravating hypoxia and impairing tubular flow dynamics [27].

### 3.3. Oxidative Stress

Administration of RCM leads to the production of reactive oxygen species (ROS), further worsening injury to tubular and endothelial cells [23].

The imbalance between ROS production and antioxidant defense systems leads to lipid peroxidation, DNA damage, and mitochondrial dysfunction [28,29]. Oxidative stress also amplifies local inflammation and endothelial dysfunction, creating a self-perpetuating cycle of injury.

### 3.4. Inflammatory and Endothelial Responses

There is evidence that suggests the role of inflammation in the pathogenesis of CI-AKI. Contrast agents stimulate the release of pro-inflammatory cytokines such as TNF-α (Tumor Necrosis Factor α) and interleukin-6 (IL-6), activation of neutrophils, and upregulation of adhesion molecules in the renal vasculature [30]. A possible role of Interleukin-8 (IL-8), a neutrophil chemoattractant, has also been implicated [31].

The resulting endothelial dysfunction increases vascular permeability and worsens renal hypoperfusion.

### 3.5. Synergistic Mechanisms

Ultimately, CI-AKI results from the synergistic effects of ischemia, cytotoxicity, and oxidative stress, leading to tubular cell injury, necrosis, and cast formation within the nephron. The resultant decrease in glomerular filtration rate (GFR) manifests clinically as an acute rise in sCr within 48–72 h of RCM exposure [3,15,32,33].

## 4. Risk Factors for CI-AKI

The development of CI-AKI depends on the interaction between patient-related (intrinsic) and procedure-related (extrinsic) risk factors (Table 2). A clear understanding of these determinants is crucial for identifying high-risk patients and implementing preventive measures prior to RCM exposure [1,15,32,34].

### 4.1. Pre-Existing CKD

Baseline renal impairment is the most significant and well-established risk factor for CI-AKI. Patients with an estimated GFR (eGFR) below 60 mL/min/1.73 m^2^, particularly those below 30 mL/min/1.73 m^2^, are at the highest risk [20,35]. The impaired autoregulatory capacity of diseased kidneys makes them more susceptible to hemodynamic changes, oxidative stress, and direct tubular toxicity from RCM. Moreover, the reduced ability to excrete the agent prolongs renal tubular exposure, thereby exacerbating injury [14].

It should be noted that, throughout this manuscript, eGFR refers to the use of various formulas to calculate it, reflecting the substantial heterogeneity among the studies included. This variability constitutes a limitation in terms of standardization and comparability of results.

### 4.2. Diabetes Mellitus

Diabetes mellitus—especially when associated with nephropathy—further increases the risk of CI-AKI [28,36]. Diabetic kidneys exhibit microvascular damage, endothelial dysfunction, and heightened oxidative stress, which predispose them to ischemic injury. Hyperglycemia also enhances tubular workload and promotes pro-inflammatory cytokine release [37].

### 4.3. Volume Depletion and Hypotension

Intravascular volume depletion, from dehydration, diuretics, or blood loss, predisposes to renal hypoperfusion and medullary ischemia. Hypotension further impairs renal blood flow, compounding ischemic injury [38]. Optimal hydration has been consistently shown to be the most effective preventive measure against CI-AKI [39,40].

### 4.4. Congestive Heart Failure and Reduced Cardiac Output

Heart failure and low cardiac output states decrease renal perfusion and oxygen delivery, amplifying susceptibility to ischemic injury [41]. Elevated central venous pressure can also impede renal venous drainage, worsening medullary hypoxia.

### 4.5. Advanced Age

Renal aging is associated with structural and functional declines—glomerulosclerosis, tubular atrophy, and reduced GFR—that heighten CI-AKI risk. Elderly patients also tend to have more comorbidities and polypharmacy, which further compound the risk [42].

### 4.6. Anemia and Hypoxemia

Low hematocrit and hypoxemic conditions reduce renal oxygen delivery, aggravating medullary hypoxia after RCM exposure [43]. Several studies suggest that anemia independently predicts CI-AKI, particularly in patients undergoing percutaneous coronary intervention [28].

### 4.7. High Contrast Volume and Type of RCM

The total RCM volume is a strong, modifiable procedural risk factor. The risk rises exponentially when the volume-to-creatinine clearance ratio exceeds 3.7 [44]. High-osmolar RCM have historically been associated with a higher CI-AKI incidence, while low- or iso-osmolar RCM significantly reduce, but do not eliminate, the risk [44,45,46]. The viscosity of the RCM is also a consideration [46], as more viscous media are eliminated more slowly and therefore result in the tubules being exposed to them for longer periods.

### 4.8. Repeated or Close Intervals of RCM Administration

Repeated exposure within 48–72 h limits renal recovery time and markedly increases CI-AKI risk, especially in patients with underlying CKD [47]. Frequent recurrence is, in fact, an independent factor even when adjusting for total volume, eGFR and comorbidities [2]. For this reason, some organizations (ESUR, European Society of Urogenital Radiology) recommend avoiding the re-administration of RCM within 4 h if the eGFR is >30 mL/min/1.72 m^2^ and waiting at least 48 h if the eGFR is <30 mL/min/1.72 m^2^ or in patients on dialysis with residual renal function [48].

### 4.9. Concomitant Nephrotoxic Medications

Concurrent use of nonsteroidal anti-inflammatory drugs (NSAIDs), aminoglycosides, cyclosporine, or loop diuretics can potentiate renal injury by impairing autoregulation, reducing renal blood flow, or causing direct tubular toxicity [49].

### 4.10. Procedural Factors

Procedures associated with prolonged ischemia—such as complex coronary interventions, use of intra-aortic balloon pump, or embolic complications—increase CI-AKI risk by combining RCM toxicity with hemodynamic stress [50].

## 5. The Relationship Between RCM and Kidney Disease Biomarkers

The effects of contrast agents on the levels of renal biomarkers represent a critical area of research and clinical application, particularly for understanding the mechanisms, early detection, and progression of CI-AKI. Contrast agents can significantly influence biomarker levels, thereby affecting their utility in the early diagnosis, risk stratification, and prognostic assessment of CI-AKI. Both traditional and emerging biomarkers play an essential role in advancing these diagnostic and therapeutic strategies [46] (Table 3).

### 5.1. Traditional Functional Biomarkers and Their Limitations

#### 5.1.1. Serum Creatinine (sCr)

sCr is among the most widely employed biomarkers in clinical practice and is fundamental for assessing and managing renal function. As a functional indicator, sCr provides an indirect estimation of GFR [47]. However, its clinical application is limited by several well-known drawbacks [48]. Increases in sCr become apparent only after considerable renal damage has occurred, with nearly 50% of nephron function already lost by the time measurable elevations are detected, thus rendering it a late marker of kidney injury [49,50,51]. Moreover, sCr levels are affected by nonrenal factors such as age, sex, muscle mass, and hydration status, potentially leading to misinterpretation of kidney function. These limitations highlight the need for more accurate and sensitive biomarkers capable of detecting renal injury at an early stage [52,53,54]. Despite these shortcomings, sCr remains a commonly used biomarker in the diagnosis of CI-AKI, although its sensitivity and specificity are suboptimal.

#### 5.1.2. Glomerular Filtration Rate (GFR)

GFR is a central parameter for evaluating renal function, as it reflects the amount of blood filtered by the glomeruli each minute. Clinically, GFR is widely applied as a standard tool for assessing kidney function and plays a crucial role in the diagnosis and classification of CKD and AKI, counting CI-AKI [55]. A key strength of GFR lies in its ability to detect early changes in renal function, often preceding the development of clinical manifestations [56]. Nonetheless, estimated GFR is subject to limitations similar to those of serum creatinine, as it is influenced by patient-related factors such as age, sex, and muscle mass.

### 5.2. Early Functional Biomarkers

#### Cystatin C (CysC)

CysC is an increasingly recognized biomarker for the early assessment of declining renal function, particularly in the setting of CI-AKI. This low-molecular-weight protein is produced by all nucleated cells, freely filtered by the glomeruli, and almost completely reabsorbed and catabolized by the renal tubules. These characteristics make CysC highly sensitive to subtle changes in GFR, often allowing earlier detection of renal injury than sCr [57].

In CI-AKI, exposure to iodinated RCM induces renal vasoconstriction and tubular toxicity, resulting in decreased glomerular filtration and impaired tubular reabsorption. Consequently, circulating CysC levels increase while urinary concentrations decline, providing an early indication of renal injury compared with sCr [48]. Unlike sCr, plasma CysC concentrations are minimally affected by age, sex, or muscle mass, making it a more accurate marker of kidney function across diverse patient populations [58].

CysC has proven its clinical value for the early diagnosis of CI-AKI, particularly when a rise of at least 15% in serum levels within 24–48 h is used as a diagnostic threshold [59]. Recent evidence suggests that CysC measured at 24 h is the most effective biomarker for diagnosing CI-AKI, whereas baseline levels of other commonly assessed biomarkers—such as interleukin-18 (IL-18), β2-microglobulin (β2M), and tumor necrosis factor-α (TNF-α)—may serve as stronger predictors of prognosis [60].

### 5.3. Biomarkers of Tubular Injury: Early Detection of Structural Damage

#### 5.3.1. β-2 Microglobulin (β-2M)

β-2M is a low-molecular-weight protein that constitutes an essential component of the major histocompatibility complex class I molecule. Under normal physiological conditions, β-2M is freely filtered by the glomeruli and almost completely reabsorbed in the proximal renal tubules. Tubular injury impairs this reabsorptive process, resulting in elevated β-2M concentrations in both serum and urine. Owing to these characteristics, β-2M has attracted increasing attention as a potential biomarker for kidney diseases, particularly CI-AKI [61,62,63,64].

Evidence suggests that serum β-2M is a strong predictor of CI-AKI. Li et al. reported that serum β-2M demonstrated significantly greater predictive accuracy than sCr, with area under the curve (AUC) values of 0.842 (*p* < 0.001) at 24 h and 0.937 (*p* < 0.001) at 48 h, compared with lower AUC values for sCr (0.691 and 0.908, respectively). These findings support the superior performance of β-2M as an early predictor of CI-AKI relative to traditional renal function markers [61].

Baseline serum β-2M levels have also been identified as independent predictors of CI-AKI. In a study by Nozue et al., a serum β-2M cutoff value greater than 2.8 mg/L achieved a sensitivity of 75% and a specificity of 80% for predicting CI-AKI. This suggests that β-2M may be a valuable early screening biomarker for identifying patients at increased risk of CI-AKI before contrast exposure [62].

Beyond its predictive role, β-2M also serves as a prognostic marker for renal recovery and disease progression. Studies have shown a marked increase in urinary β-2M levels following exposure to radiographic contrast media, reflecting acute tubular injury. In animal models, urinary β-2M concentrations increased up to 126-fold after contrast administration [63]. Comparable trends have been observed in human studies, although statistical significance remains limited [62]. Overall, these findings suggest that urinary β-2M primarily reflects acute tubular damage, whereas serum β-2M may provide a more stable and reliable indicator of renal dysfunction over time.

#### 5.3.2. Osteopontin (OPN)

OPN is a pleiotropic glycoprotein involved in a wide array of physiological and pathological processes that plays a role in immune regulation, cell adhesion, and tissue remodeling. Its expression is upregulated in response to tubular injury induced by oxidative stress and hypoxia, highlighting its potential utility as a biomarker for CI-AKI. Elevated OPN levels are closely associated with inflammatory processes, tubular damage, and fibrotic changes, supporting its role as an early indicator of renal injury [65,66,67].

#### 5.3.3. Kidney Injury Molecule-1 (KIM-1)

KIM-1 is widely regarded as a signature biomarker for tubular injury, offering superior specificity in identifying a localized renal insult and demonstrates significant potential for the early detection of CI-AKI. This type I transmembrane glycoprotein is minimally expressed in healthy kidneys but is markedly upregulated in proximal tubular cells following nephrotoxic injury [55,68,69,70]. Exposure to RCM induces oxidative stress, renal medullary hypoxia, and tubular epithelial cell damage, which in turn promotes the release of KIM-1 into the urine, serving as a non-invasive indicator of tubular injury. Urinary KIM-1 levels typically rise before changes in serum creatinine, often as early as six hours after angiographic procedures, offering a valuable window for early diagnosis [71,72,73].

#### 5.3.4. Neutrophil Gelatinase-Associated Lipocalin (NGAL)

NGAL is well-established as a highly sensitive biomarker for the early identification of renal insults, especially when secondary to ischemic or toxic triggers [74,75]. NGAL is expressed in the ascending loop of Henle and the collecting ducts—regions susceptible to injury from iodinated contrast agents. Following a renal insult, NGAL may be released and detected in both urine and plasma within 2–6 h [70,76]. While NGAL is highly sensitive to tubular damage, it lacks specificity for CI-AKI, as its levels may also rise in conditions such as sepsis, systemic infections, and CKD [54].

### 5.4. Inflammatory and Oxidative Stress-Related Biomarkers

#### IL-18 (Interleukin-18)

IL-18 is a pro-inflammatory cytokine belonging to the interleukin-1 superfamily and plays a key role in immune regulation and tissue inflammation. It is considered a marker of tubular inflammation and an early indicator of AKI, as tubular damage caused by oxidative stress and hypoxia triggers the release of IL-18 from renal proximal tubular epithelial cells [47,55].

In CI-AKI, exposure to RCM promotes ROS generation and renal medullary hypoxia, leading to activation of inflammatory pathways. As a result, IL-18 is released and can be detected in both urine and serum. Elevated urinary IL-18 levels correlate with the severity of tubular injury, supporting its role as a valuable biomarker for early diagnosis. IL-18 demonstrates high specificity for acute tubular inflammation and, unlike sCr, which rises later during kidney injury, IL-18 levels increase early, often within hours of a renal insult [77,78,79,80,81].

A study by Zdziechowska et al. evaluated patients undergoing elective or emergency coronary angiography with contrast administration and measured serum IL-18 concentrations at baseline, 24 h, and 72 h after the procedure. The results showed increased IL-18 levels in patients who developed CI-AKI, reaching near statistical significance at 24 h; however, levels declined by 72 h, suggesting limited reliability of serum IL-18 as a sustained biomarker for CI-AKI [78].

IL-18 is commonly measured using ELISA or Bio-Plex technology. A urinary IL-18 increase of more than 25% at 24 h following contrast exposure has been proposed as a diagnostic threshold for CI-AKI.

### 5.5. Other Biomarkers

Numerous other biomarkers have been and are being studied for the early diagnosis of CI-AKI. These are molecules with different origins: tubular damage, inflammatory oxidative stress, cell damage and apoptosis, endothelial dysfunction, renal fibrosis, systemic markers and other traditional markers [51]. The employment of new biomarkers in clinical practice for the prevention and management of CI-AKI represents a very promising strategy. It could enable the early application of preventive measures to reduce risk as much as possible. Furthermore, it could allow for the identification of patients at higher risk, enabling clinicians to correct or eliminate the use of nephrotoxic agents. However, addressing challenges associated with cost, standardization, and clinical implementation is essential for their broader acceptance [37].

## 6. Prevention

The incidence of CI-AKI is higher in cases of arterial RCM administration compared with venous administration. This difference is only partly related to the enhanced nephrotoxicity of intra-arterial RCM and is mainly linked to the risk of cholesterol embolization in procedures involving intra-arterial administration as well as to the intrinsic characteristics of the population (in fact, patients undergoing angiographic procedures often have greater comorbidities that expose them to a higher risk of AKI). This difference requires different prophylactic strategies for angiographic procedures and CT scanning with intravenous RCM.

The main preventive measures are summarised in Table 4.

### 6.1. Prevention Measures for Intra-Arterial RCM Administration

Generally, preventive measures are used in all patients undergoing intra-arterial RCM administration [52,53,54,55], although high-risk categories can be identified such as: significant proteinuria (proteinuria > 500 mg/day or albuminuria > 300 mg/day) regardless of eGFR; patients with an eGFR < 60 mL/min/1.73 m^2^ and specific comorbidities (multiple myeloma, diabetes mellitus, heart and liver failure); patients with an eGFR < 30 mL/min/1.73 m^2^ regardless of the presence of proteinuria and/or comorbidities.

Specific preventive measures are typically recommended and include:

- Both volume depletion and the administration of nephrotoxic drugs should be avoided [56], particularly NSAIDs, aminoglycosides, amphotericin B and some antivirals (foscarnet, acyclovir). Both volume depletion and NSAIDs, for example, induce intrarenal vasoconstriction.
- Choice of RCM type and dose: As recommended by various guidelines [57,58], low-osmolar (such as iodixanol) or nonionic iso-osmol agents (such as iopamidol) should be preferred over high-osmolarity RCM. Furthermore, the lowest possible dose should be used, and closely spaced contrast studies (within 48–72 h) should be avoided [59,60,61,62,63].

A broader discussion is needed on the main preventive measure, namely fluid administration. Intravenous fluid administration is the most widely used, effective and safe method of prophylaxis for the prevention of CI-AKI [64]. There are several reasons why fluid administration is effective:

(i) Maintaining euvolemia prevents hyperactivation of the sympathetic nervous system and the RAAS, counteracting renal vasoconstriction caused by the RCM and thus renal hypoperfusion;
(ii) The dilution of the RCM within the renal tubular lumen reduces its contact time with renal tubular cells and consequently its direct tubular toxicity [44,77].

It has long been recognized that oral fluid administration alone is insufficient to adequately prevent this risk, and therefore intravenously infused solutions must be used [78] although there are no well-designed randomized controlled trials to demonstrate this benefit. Data from randomized trials regarding the sole effect of fluid therapy remain limited, as it is rarely studied independently of additional treatments like forced diuresis [38,65,79,80,81]. Some of these have demonstrated benefits with intravenous fluid administration, but they were certainly not free from some significant biases. In two trials involving 408 and 216 patients (most with normal renal function) with acute myocardial infarction undergoing percutaneous coronary intervention (PCI), intravenous administration of saline solution resulted in a reduction in the risk of CI-AKI [65,81]. Furthermore, one of the two trials demonstrated a statistically significant reduction in in-hospital mortality among patients receiving fluid therapy [81]. In both studies, the incidence of CI-AKI (presumably reduced by fluid administration) may have been influenced by the prevention of hypotensive events rather than by a direct protective effect of fluids against CI-AKI. Another trial with a very small sample of patients [80] undergoing cardiac catheterisation (non-emergency) showed that intravenous infusion of saline solution reduced the incidence of CI-AKI compared with the group undergoing oral hydration. The study was stopped early due to the effectiveness of the intervention, and also because hyperosmolar ionic RCM were used, which are considerably more nephrotoxic than those commonly used today.

It is also worth mentioning a study that showed conflicting results compared with the trials described above. This was a single-centre randomised trial (AMACING) involving a group of patients with an eGFR between 30 and 59 mL/min/1.73 m^2^ and no particular risk factors for CI-AKI, in which intravenous administration of saline solution showed no benefit in reducing the risk of CI-AKI [38]. In addition, some adverse events occurred in the treatment group, including arrhythmias, heart failure and hyponatraemia. In a comprehensive hierarchical Bayesian network meta-analysis of 124 randomized trials encompassing over 28,000 patients undergoing PCI, ten distinct preventive strategies were evaluated and compared. This meta-analysis found that, compared to saline alone, six other strategies (statins, xanthine, ischaemic preconditioning, N-acetylcysteine (NAC), sodium bicarbonate, and NAC + sodium bicarbonate) were more effective in reducing the risk of CI-AKI, with greater benefits than statin therapy [66]. It is worth noting that the study found hydration alone to be the most ineffective preventive strategy. Notably, no specific preventive measure proved effective in lowering CI-AKI rates within the subgroup of patients with diabetes mellitus. The strengths of this analysis are the large number of studies included and, above all, the attempt not to limit itself to comparing two preventive strategies. However, the study included clinical trials with highly variable sample sizes, highly variable baseline sCr values, and an extremely variable patient population [67]. All of this severely limits the results of this study.

Several hydration protocols have been studied and are used in clinical practice in various hospitals. However, to date, no definitive consensus exists on how hydration for the prevention of CI-AKI should be carried out. The European Society of Cardiology (ESC)/European Association for Cardio-Thoracic Surgery (EACTS) [68] guidelines and the Kidney Disease: Improving Global Outcomes (KDIGO) guidelines [69] both recommend a 1 mL/Kg/hour infusion of normal saline 12 h before and 12 h after RCM administration. Nonetheless, several recent studies have reinforced the concept of personalised hydration protocols: a single hydration protocol should not be applied to all patients. There are several factors to consider, primarily including a patient’s characteristics, such as cardiac function, fluid and electrolyte balance, acid-base balance and comorbidities, as well as the risks of fluid overload. This has led to the need to develop a new approach based on the concept of controlled hydration [70]. It is widely recognized that fluid overload can exacerbate the risk of heart failure, arrhythmias, and short-term mortality, particularly among high-risk patient populations. Possible ways of implementing a controlled hydration strategy could include the use of haemodynamic parameters such as central venous pressure (CVP), left ventricular end-diastolic pressure (LVEDP) [71], vena cava collapsibility [72], bioimpedance and urinary volume [73].

Notably, a randomised, non-placebo-controlled study (POSEIDON) demonstrated (indirectly) a positive effect of fluid administration depending on its dose [71]. In this study, which included patients with eGFR < 60 mL/min/1.73 m^2^ and other risk factors, an aggressive fluid replacement protocol guided by LVEDP was applied and compared with the standard dose of intravenous hydration. All patients received an intravenous infusion of isotonic saline solution at rate of 3 mL/Kg for one hour prior to the procedure, and LVEDP was measured before administration of the contrast agent. In the LVEDP group, the hydration protocol was guided by LVEDP levels and, in particular:—if LVEDP was less than 13 mmHg, fluids were administered at a dose of 5 mL/Kg; if LVEDP was between 13 and 18 mmHg, fluids were administered at a dose of 3 mL/Kg; if it was above 18 mmHg, fluids were administered at a dose of 1.5 mL/Kg. Finally, patients in the control group received fluids at a dose of 1.5 mL/Kg. The solution was started during the procedure and continued for the following four hours. Patients in the LVEDP-guided hydration group experienced a significantly lower incidence of CI-AKI compared with the control group (6.7% vs. 16.3%, respectively).

An interesting system capable of coordinating hydration demand and the risk of overload is RenalGuard (RenalGuard Solutions, Milford, Massachusetts, USA), a device that optimizes volume expansion by matching the infused volume to urinary output (urine flow rate [UFR]-guided hydration) [74]. Priming of the system is followed by a bolus of saline solution (approximately 3 mL/Kg in 20–30 min) and furosemide (0.25 mg/Kg), thereby maintaining high urine output to rapidly eliminate the contrast agent, protecting the tubules from its harmful effects. The first dose of RCM is infused as soon as urine output reaches 300 mL/h. Randomised trials have already demonstrated that the use of this system of forced diuresis with furosemide and hydration, compared with a fixed hydration regimen, can reduce the risk of CI-AKI and the need for renal replacement therapy [75]. As already mentioned, one of the primary considerations in pre-anaesthetic hydration is to assess the patient’s fluid status to prevent overhydration. In this regard, a research group has demonstrated that this system is also effective in reducing the risk of pulmonary edema [76]. An Italian multicentre study (REMEDIAL II) conducted by the same group also demonstrated that the RenalGuard system is superior even to therapy with NAC and sodium bicarbonate in reducing the incidence of CI-AKI in high-risk patients [82]. Others have obtained very similar results [83,84]. A meta-analysis was also conducted on this system to clarify its potential benefits and demonstrated not only a reduction in the rate of CI-AKI but also in cardiovascular events and mortality [85]. Recently, a meta-analysis was published that highlighted the benefits of reducing the incidence of CI-AKI with the use of the RenalGuard system in moderate-to-high-risk populations and CKD patients subjected to cardiac surgery. However, it failed to demonstrate a statistically significant impact on mortality rates, the need for dialysis, pulmonary edema, major cardiovascular events, and sCr levels compared with the control group [86].

Furthermore, a group of Italian researchers recently conducted an analysis to determine the effective volume of intravenous fluids required to reduce the risk of CI-AKI [87]. This absolute volume was found to be equal to or greater than 1460 mL, while the ratio between the expansion volume and that of the RCM was found to be equal to or greater than 17; these were the dichotomous values in reducing the risk of CI-AKI.

Based on current evidence, isotonic saline is generally recommended as the first-line fluid for CI-AKI prevention given its consistent safety profile and supporting evidence, although no strategy has demonstrated clear superiority across all patient populations and alternative strategies should be tailored to individual patient risk.

#### 6.1.1. Types of Solutions Used for the Prevention of CI-AKI

There is strong evidence demonstrating that isotonic saline solutions are superior to hypotonic ones [35,88]. In a randomised trial, hypotonic saline solution was compared with isotonic saline solution, and the latter appeared to reduce the risk of CI-AKI (2% vs 0.7%) [88]. Notably, no significant difference was observed within the subgroup of patients exhibiting moderate-to-severe renal impairment (sCr > 1.6 mg/dL), which represents an important limitation of this study, as individuals with renal dysfunction are at greater risk of further deterioration of renal function following RCM administration. The greatest benefits were seen in those who received an RCM infusion of more than 250 mL and in diabetic subjects.

Isotonic saline solutions and those containing bicarbonate have both been found to be effective. However, there does not seem to be any substantial difference between the two except that bicarbonate is more expensive. Several randomised controlled trials and meta-analyses have compared the two solutions in this matter, but the results have been conflicting [89,90,91,92,93,94,95,96,97,98,99,100,101,102,103]. A relatively recent randomised trial (PRESERVE) demonstrated that both solutions are similarly effective [104]. A total of 4993 high-risk patients were recruited for elective angiography, 81% of whom were diabetic. Patients were prescribed 1.26% sodium bicarbonate or saline solution (0.9% sodium chloride) and NAC orally (a loading dose of 1200 mg was administered one hour prior to and one hour following angiography, followed by a maintenance regimen of 1200 mg twice daily for four days) or corresponding placebo capsules. The infusion protocol stipulated that the solutions be administered at a total dose of 3 to 12 mL/Kg before the procedure (one to 12 h beforehand), 1 to 1.5 mL/Kg during the procedure, and a total dose of 6 to 12 mL/h after the procedure (6 to 12 h afterwards). The analyzed outcomes showed similar incidence between the two groups (AKI, need for dialysis, persistence of renal damage at 90 days and death).

However, few data are available on the effectiveness of preventing CI-AKI with the use of Ringer’s solution compared with sodium saline solution [105].

#### 6.1.2. Other Tested Prevention Strategies

##### Oral Salt Loading

There is no solid evidence regarding oral salt loading. A randomised trial showed that sodium loading (1 g/10 Kg orally) in the two days prior to the procedure was as effective as intravenous hydration [106]. Similar results were also obtained in another study in which a combined hydration protocol was used in outpatients, consisting of oral hydration followed by a short period of intravenous hydration [107].

##### NAC

Among all the molecules tested for RCM prophylaxis, NAC certainly stands out. It is an important reducing agent containing a sulphydryl group, known for its remarkable antioxidant properties capable of promoting glutathione synthesis and regulating cellular metabolism.

Moderate benefits were reported in meta-analyses that failed to adjust for the significant heterogeneity observed across the included studies [108]. Nevertheless, notwithstanding numerous meta-analyses conducted on the use of this molecule in the prevention of CI-AKI, the results have often been conflicting [52,96,104,109,110,111,112,113,114,115,116,117,118,119,120,121,122,123,124,125,126]. Following these meta-analyses, a large, randomised trial (PRESERVE) was published involving 4993 high-risk patients undergoing elective angiography, which showed no benefits with the use of NAC [104]. No statistically significant difference was observed between the placebo and NAC prophylaxis groups in terms of AKI, the need for dialysis, and impaired renal function at 90 days. In addition, several adverse events have been reported in some studies with intravenous administration [127,128,129], including anaphylactic reactions [128].

##### Statins

Hypercholesterolaemia could be a risk factor for RCM-induced kidney damage, as shown by experimental models of AKI, characterised by impaired NO synthesis and increased ROS production [130]. In fact, in addition to their well-known lipid-lowering action, they exert anti-inflammatory effects by inhibiting the production of ROS and scavenging free radical species [131]. Furthermore, they promote the activation of endothelial progenitor cells, thereby enhancing vascular protection and repair [132]. Some studies [133,134,135,136,137,138,139] but not all [140], have suggested that statins may exert protective effects, potentially reducing the incidence of CI-AKI. An analysis encompassing five trials found that triple therapy—consisting of statins, NAC, and intravenous saline—was more effective in mitigating CI-AKI risk than the dual NAC-saline approach [141]. However, a meta-analysis encompassing eight studies (n = 5024) failed to demonstrate a statistically significant benefit of statin therapy as an adjunct to intravenous saline compared with saline hydration alone [89]. Overall, the literature supports the beneficial pleiotropic effects of statin use on CI-AKI, although further randomised clinical trials are needed to confirm the actual benefits [142]. Statins are certainly indicated for use in patients with acute myocardial infarction before undergoing angiography.

##### Diuretics

Diuretics are indeed fully indicated in the treatment of volume overload. However, they should not be started solely for the prophylaxis of RCM-induced kidney damage, as they do not appear to provide any benefit [143,144,145]. Forced diuresis combined with proportionate volume replacement reduces the incidence of AKI after angiography compared with traditional hydration protocols [82,84,146].

##### Ascorbic Acid

Studies evaluating a specific oral dosing schedule of ascorbic acid (3 g pre-angiography and 2 g twice post-angiography) have failed to reach a consensus, providing contradictory evidence regarding its protective effect [147,148]. Only one study has highlighted the potential benefits of using ascorbic acid to reduce the risk of CI-AKI [147]. However, a meta-analysis (of 6 studies) did not highlight any benefits in the prophylactic use of this molecule [89].

##### Vitamin E (α- or γ-Tocopherol)

Numerous studies (both in vitro and in vivo) have highlighted the anti-inflammatory and antioxidant effects of vitamin E [149]. Regarding CI-AKI prophylaxis in CKD patients undergoing elective coronary angiography, the efficacy of oral α- and γ-tocopherol—administered from five days pre-procedure to two days post-procedure—was evaluated in conjunction with saline infusion. [150]. The authors found a reduction in the risk of CI-AKI in the treatment group.

##### Nebivolol

Nebivolol is a third-generation β1-selective adrenergic receptor antagonist characterized by its unique vasodilatory and antioxidant properties. [151,152]. The use of nebivolol in the prophylaxis of CI-AKI stems from its antioxidant actions and, above all, from NO-induced vasodilation. The use of nebivolol (at a dose of 5 mg per day for one week) was studied in a group of patients with renal dysfunction undergoing coronary angiography, and it reduced the incidence of renal injury [153]. However, the sample size was very small, so it is not possible to reach a definitive conclusion. Very similar results (again on a small number of patients) were obtained from another study conducted later [154].

##### Atrial Natriuretic Peptide

Atrial natriuretic peptide (anaritide, ANP 4–28) has been tested on animals (canine models) in the prevention of CI-AKI with favourable results [155]. Also, given the promising results from in vitro studies, a randomised, double-blind, controlled trial was subsequently conducted to test the use of ANP in patients with (stable) renal dysfunction undergoing iodinated RCM administration. However, no statistically significant difference in the incidence of CI-AKI was observed [156].

##### Inorganic Nitrate

It is known that the nephrotoxicity of iodinated RCM may also be linked to ischaemia caused by vasoconstriction and ROS resulting from reduced NO availability.

In a trial (NITRATE-CIN) involving 640 patients undergoing angiography, the use of oral inorganic nitrates was tested [157]. The preventive use of these drugs proved effective in reducing the risk of cardiovascular events and CI-AKI.

##### Sodium Citrate

Another molecule that has been tested is oral sodium/potassium citrate (Na/K citrate). Notably, a randomized controlled trial demonstrated that urinary alkalinization—achieved via Na/K citrate administration (5 g in 200 mL, one hour prior to and four hours following angiography)—could effectively mitigate the risk of CI-AKI [158]. Similar results were obtained very recently in a prospective randomised study in which Na/K citrate was also used (2 h before and 4 h after elective coronary angiography) in combination with hydration [159]. Therefore, it could be a promising molecule for preventing CI-AKI if further studies are conducted to confirm these initial observations, especially with larger patient samples.

##### Agents Preventing Vasoconstriction

Numerous molecules preventing vasoconstriction (such as fenoldopam, iloprost, nifedipine, low-dose dopamine, captopril, theophylline or aminophylline, prostaglandin E or I2) [160,161,162,163,164,165] have been checked for the prevention of CI-AKI with contrasting results. Some of these studies have demonstrated potential beneficial effects of vasodilator use [160,161,162], while others have not [163,164,165]. Therefore, it is currently not possible to make definitive judgments about these molecules.

##### Novel Antidiabetic Agents

In recent years, new hypoglycaemic agents have been introduced, some of which have had surprisingly beneficial effects on cardiovascular and renal outcomes and are also revolutionising the treatment of CKD.

Recently, a group of Italian researchers analysed the effect of three new classes of hypoglycemic agents on the risk of CI-AKI in diabetic patients undergoing PCI [166]. A group of 293 diabetic patients was prescribed with one of three classes of oral hypoglycemic agents: glucagon-like peptide-1 receptor agonists (GLP-1RAs), sodium-glucose transporter-2 inhibitors (SGLT2i), and dipeptidyl peptidase-4 inhibitors (DPP4i). The use of these agents is associated with a lower incidence of renal damage in diabetic patients undergoing PCI, with the greatest benefit observed with the use of SGLT2i and GLP-1RAs. Moreover, a recent registry cross-sectional study revealed that Dapaglifozin has robust cardioprotective effects against periprocedural myocardial injury (PMI) with or without type 4a myocardial infarction (4aMI) in patients with type II diabetes mellitus and acute coronary syndrome undergoing PCI [167]. However, the drug did not improve renal outcomes (risk of CI-AKI). These results highlighted the cardiovascular benefits of these drugs even in this high-risk patient category. Conversely, some evidence suggests that Empagliflozin exerts a protective effect against CI-AKI in patients undergoing PCI (regardless of whether they were diabetic or not) in terms of eGFR and CysC [168]. The best results were obtained in middle-aged and elderly subjects, regardless of renal function values.

##### Oxygenation

Recently, a group of Indian researchers evaluated the effectiveness of oxygenation in preventing CI-AKI in patients with CKD stage 3–5 undergoing coronary angiography [169]. Oxygen supplementation (2 L/min) and saline hydration effectively prevented CI-AKI. These are preliminary data, but they could serve as a starting point for subsequent larger-scale randomised trials.

##### Remote Ischemic Pre-Conditioning

Remote ischemic preconditioning (RIPC) is a strategy in which the induction of brief, non-lethal ischemia in a localized organ or tissue confers systemic protection against subsequent ischemic injury in a distant organ. This is a fascinating approach, and over the last decade there has been some evidence of its possible protective effect against CI-AKI [170,171], with very recent reports confirming these findings [172,173]. In particular, a randomised multicentre study involving patients at high risk of CI-AKI undergoing elective coronary angiography or PCI demonstrated that the incidence of CI-AKI was lower in patients receiving delayed RIPC compared with sham RIPC [172]. Similar data were collected in a randomised trial involving a cohort of patients undergoing high-risk elective coronary angiography, which showed not only a reduction in CI-AKI but also a reduction in the incidence of major cardiovascular events and an improvement in biomarkers of kidney damage such as *t*issue inhibitor of metalloproteinases 2 (TIMP2) and *i*nsulin-like growth factor-binding protein-7 (IGFBP7), two markers of AKI [173]. Despite these promising preliminary data, further confirmation from robust clinical trials is required to establish this approach as a standard preventive measure for CI-AKI.

##### Enhanced External Counterpulsation

Enhanced External Counterpulsation (EECP) is a non-invasive, outpatient therapy that treats chronic stable angina and heart failure by using external cuffs to increase blood flow to the heart [174]. This therapy utilizes pneumatic cuffs applied to the lower extremities and buttocks, which inflate and deflate sequentially in synchrony with the cardiac cycle. By optimizing diastolic augmentation, EECP enhances coronary perfusion and systemic oxygen delivery, thereby alleviating anginal symptoms and improving the overall quality of life. Furthermore, there is evidence to suggest that EECP significantly increases renal blood flow [175], attenuates renal injury, and promotes endothelial vasodilation by shifting the balance between plasma NO and endothelin-1 concentrations [176]. A recent study conducted in China demonstrated that EECP significantly improved eGFR and mitigated the risk of CI-AKI among diabetic patients with CKD undergoing coronary angiography or PCI [177].

##### Withholding ACE Inhibitors and/or Angiotensin II Receptor Blockers (ARBs)

RAAS inhibitors are unquestionably one of the most widely used antihypertensive drugs with proven renal and cardiovascular benefits, particularly in patients with kidney disease. They cause vasodilatation of the efferent renal arterioles and decrease the intraglomerular pressures [178]. Their nephroprotective effect is mainly due to this effect. RAAS inhibitors may also have a protective role by inhibiting RCM-induced vasoconstriction of the afferent arteriole. Not only that, but angiotensin II inhibition also leads to a reduced production of ROS and an increased production of NO [179,180]. However, it is well established that ACE inhibitors suppress the synthesis of transforming growth factor-beta 1 (TGF-β1) [181] and the latter appears to play a protective role against damage (and necrosis) to proximal tubular cells [182]. This could be one of the possible reasons why ACE inhibitors and ARBs may exacerbate the effects of RCM.

Indeed, it has been found that patients using ACE inhibitors and ARBs have an increased risk of kidney damage after exposure to iodinated RCM [183]. Similar results were observed in other randomised trials, both in a population of patients with sCr between 1.5 and 3.5 mg/dL undergoing left heart catheterisation [184] and in elderly patients with mild to moderate CKD undergoing elective coronary angiography [185]. Retrospective studies [183,186] have also shown a higher incidence of CI-AKI with the use of ACE inhibitors and ARBs, recommending their discontinuation 48 h prior to the angiography procedure. However, randomised clinical trials are still needed to confirm this.

Other publications have shown a neutral effect of these drugs. For instance, a study of 220 patients with CKD stages 3–4 (eGFR between 15 and 59 mL/min/1.73 m^2^) showed that discontinuing ACE inhibitors or ARBs prior to angiography does not affect the risk of CI-AKI [187].

Furthermore, other studies have even shown a potential protective effect of ACE inhibitors/ARBs with respect to CI-AKI [188,189,190].

Overall, it is currently unclear whether discontinuing ACE inhibitors and ARBs prior to an RCM-enhanced examination can reduce the risk of CI-AKI.

##### Prophylactic Hemodialysis and Hemofiltration

The latest KDIGO guidelines on AKI do not recommend the prophylactic use of intermittent hemodialysis (HD) or hemofiltration (HF) for the removal of iodinated RCM in patients at high risk of CI-AKI [57].

Although hemodiafiltration (HDF)/HF and high flux-HD remove contrast media more effectively than low-flux HD and HF during each treatment session, their ability to prevent CI-AKI remains controversial [191,192,193,194].

A 2013 meta-analysis comprising three studies on HF/HDF and eight studies on intermittent HD did not show any benefit of renal replacement therapy for contrast agents [195]. Moreover, there is no indication to anticipate or perform additional dialysis sessions in patients already undergoing chronic HD treatment [196]. Furthermore, the same recommendation (not to perform additional sessions) also applies in cases where the objective is to preserve any residual renal function or to prevent allergic reactions (or other toxic effects) to the contrast agent in dialysis-dependent patients [196,197,198].

##### Other Potential Strategies

Other compounds/molecules have been finally investigated as potential protective strategies, to reduce renal damage in renal tubular cell lines exposed to RCM, such as Darbopoietin, quercetin and grape juice extracts [199,200,201,202]. In the case of quercetin, a phase II clinical trial was conducted whereby cardiac patients receiving RCM were treated with orally administered quercetin (500 mg/8 h; 3–5 days prior to RCM treatment). The authors reported a lower incidence of CI-AKI in the treated group [203].

### 6.2. Prevention Measures for Intravenous RCM Administration

There is a substantial difference in the incidence of CI-AKI between intra-arterial administration (angiography) and intravenous administration (CT with RCM).

In a series of retrospective cohort studies, which also included high-risk patients undergoing CT with RCM, they described a risk of CI-AKI ranging from 12 to 50% [5,32,204,205,206,207]. In some of these cohorts, no risk of CI-AKI was found even when eGFR was taken into consideration, while in others it was identified in populations with severely reduced eGFR (<30 mL/min/1.73 m^2^) [5,204,205,206,207]. In general, based on the data currently available in the literature, it can be inferred that:

(i) Patients with eGFR > 45 mL/min/1.73 m^2^ are essentially at no risk of CI-AKI;
(ii) Patients with eGFR between 30 and 44 mL/min/1.73 m^2^ have a low risk, but the incidence is difficult to assess [207];
(iii) In patients with eGFR < 30 mL/min/1.73 m^2^, the risk appears to be higher [207].

Clinically relevant nephrotoxicity in cases of CT with RCM is almost negligible in patients who are not at high risk. Those who should undergo prophylaxis are individuals who present certain risk factors, such as: -eGFR < 30 mL/min/1.73 m^2^ (not on dialysis); AKI from any other cause; eGFR between 30 and 45 mL/min/1.73 m^2^ in association with multiple comorbidities that constitute risk factors. In general, it can be inferred that no prophylactic measures are indicated in cases of stable eGFR above 30 mL/min/1.73 m^2^, in patients undergoing chronic hemodialysis and in cases of CT scans requested in life-threatening emergencies for the patient. These recommendations are also confirmed by the Consensus Statements from the American College of Radiology (ACR) and the National Kidney Foundation [2].

#### 6.2.1. Fluid Administration

The preventive measures adopted are similar to those used for angiography. However, there is no evidence from adequately designed randomised trials or observational studies demonstrating a real benefit of intravenous hydration even in cases of CT with RCM in high-risk patients [57,208]. In fact, the main evidence suggesting the use of intravenous hydration over oral hydration (or no hydration) comes from studies performed with angiography and not with intravenous RCM administration.

Three trials enrolled patients with CKD to undergo intravenous RCM administration, but none of the three included subjects with an eGFR < 30 mL/min/1.73 m^2^ or with ongoing AKI; none of the three studies demonstrated benefits with intravenous hydration in this context [38,79,209]. Recently, a large study was published with the aim of verifying the actual effectiveness of hydration in preventing CI-AKI in high-risk patients (eGFR < 30 mL/min/1.73 m^2^) undergoing intravenous administration of iodinated RCM [208]. The results indicated that periprocedural hydration—administered both before and after intravenous RCM exposure—was not associated with a reduced risk of CI-AKI, dialysis at discharge, or in-hospital mortality.

The evidence for the choice of solution to use is based on angiographic studies and therefore does not differ from what was stated earlier. Isotonic saline solutions are preferred [104], although there is some evidence that bicarbonates are equally effective, if not more so [82,89,90,91,92,93,94,95,96,97,98,99,100,102,103,104,210].

Hydration protocols often differ from centre to centre in terms of total volume, infusion rate and timing, particularly with regard to whether patients are inpatients or outpatients. In some cases, clinicians prefer oral hydration to intravenous hydration in outpatients [211].

#### 6.2.2. Withdrawal of Metformin

According to the 2018 American College of Radiology (ACR) guidelines, metformin should be discontinued either prior to or at the time of contrast-enhanced CT in patients at high risk for CI-AKI, and withheld for at least 48 h following the procedure [9]. In patients who are not at high risk, however, metformin should not be discontinued and subsequent reassessment of kidney function is not required following the procedure.

Nevertheless, the 2016 US Food and Drug Administration (FDA) guidelines advised the discontinuation of metformin in patients with an eGFR ranging from 30 to 60 mL/min/1.73 m^2^. However, there is evidence in the literature suggesting that patients taking metformin are not at high risk of CI-AKI [212].

## 7. Treatment

Although intravenous hydration is recommended for the prevention of CI-AKI by various organisations such as the 2012 KDIGO AKI Clinical Practice Guidelines [69] and the 2018 European Society of Urogenital Radiology (ESUR) CI-AKI Prevention and Treatment Guidelines [213], unfortunately there is no treatment for CI-AKI [4]. The common supportive therapies used in AKI are generally applied and is very similar to that for ischaemic/toxic AKI (such as acute tubular necrosis), including: haemodynamic optimisation, fluid management, discontinuation of nephrotoxic agents, and treatment of complications. There are no specific treatments or effective strategies to accelerate renal recovery.

## 8. Conclusions

CI-AKI remains a common and clinically significant complication, particularly in high-risk patients with CKD, diabetes mellitus, and cardiovascular comorbidities. Its pathogenesis is multifactorial, involving renal hemodynamic alterations, direct tubular toxicity, oxidative stress, and inflammatory responses.

Despite increasing knowledge of its underlying mechanisms and the emergence of novel biomarkers for earlier detection, effective management remains largely preventive. Adequate volume expansion, minimization of contrast exposure, and careful identification of at-risk patients continue to represent the most reliable strategies to reduce its incidence. Importantly, no specific treatment has been proven effective once CI-AKI has developed, and current management is limited to supportive care.

This review provides an updated and integrated overview of CI-AKI by combining recent evidence on pathophysiological mechanisms, emerging biomarkers, and evolving preventive strategies, while also emphasizing the clinical relevance of repeated contrast exposure and cumulative risk. By addressing both traditional concepts and more recent insights, this work aims to support a more individualized and evidence-based approach to patient management. Future research should focus on improving early diagnostic tools and developing targeted therapeutic strategies to mitigate renal injury and enhance recovery.

In conclusion, it can be stated that in recent years many efforts have been made to apply new drugs, some recently discovered, together with new procedures in order to reduce the incidence rate of CI-AKI. Unfortunately, the incidence is still unacceptably high and will remain so until safer RCM become available.

## Figures and Tables

**Table 1:** Some iodinated RCM that are used in clinical practice.

Name	Type	Iodine Content (mg/mL)	OSM (mOsm/kg)	Viscosity (Cps at 37 °C)
**Ionic**				
Diatrizoate (Hypaque 50)	Monomer, H	300	1550	10.5
Metrizoate (Isopaque 370)	Monomer, H	370	2100	3.4
Iothalamate (Conray)	Monomer, H	325	1843	4.0
Ioxaglate (Hexabrix)	Dimer, L	320	580	7.5
**Nonionic**				
Iopamidol (Isovue-370)	Monomer, L	370	796	9.4
Iohexol (Omnipaque 350)	Monomer, L	350	884	10.4
Iodixanol (Visipaque 320)	Dimer, I	320	290	11.8
Iotrolan (Isovist)	Dimer, I	300	320	8.1
Ioxaglate (Hexabrix)	Dimer, L	320	600	7.5
Ioxilan (Oxilan 350)	Monomer, L	350	695	8.1
Iopromide (Ultravist 370)	Monomer, L	370	774	10.0
Ioversol (Optiray 300)	Monomer, L	300	651	5.5
Iomeprol (Iomeron 350)	Monomer, L	350	618	7.5

The osmolarity of contrast media is compared with the osmolarity of plasma. H = high osmolar RCM, which have the highest osmolarity (5–8 times the osmolarity of plasma). L = low osmolar RCM, which have a lower osmolarity but still higher than plasma (2–3 times the osmolarity of plasma). I = isoosmolar RCM, which have the same osmolarity as plasma. Cps: viscosity in centipoise.

**Table 2:** Risk factors in CI-AKI.

Intrinsic Risk Factors	Extrinsic Risk Factors
Pre-existing CKD	Type of RCM/volume of RCM used
Diabetes mellitus	Repeated RCM administration
Volume depletion/hypotension	Concomitant nephrotoxic medications
Congestive heart failure/reduced cardiac output	Medical procedures associated with prolonged ischemia
Advanced age	
Anemia/hypoxemia	

**Table 3:** **Biomarkers in CI-AKI and their method of detection.**

Biomarker	Method of Efficacy as Biomarker Detection
Cystatin C (Cys-C)	Enzyme-linked early marker, Immunosorbent measured at 24 h assay (ELISA) and nephelometric and turbidometric assays
β2-Microglobulin (β2M)	ELISA and early marker; nephelometric assay detects tubular damage
Interleukin-18 (IL-18)	ELISA early marker; unreliable as a sustained biomarker
Kidney injury molecule-1 (KIM-1)	ELISA and early marker; Immunoblotting high specificity; detects tubular damage
Neutrophil gelatinase-associated Lipocalin (NGAL)	ELISA, immunoblotting, early marker; and turbidometric detects damage to assay Loop of Henle and collecting ducts
Osteopontin (OPN)	ELISA and early marker; Immunoblotting detects inflammation, tubular damage, fibrosis
Glomerular Filtration Rate (GFR)	Inulin clearance early marker; lacks sensitivity/specificity
Serum creatinine (sCr)	Blood/urine tests late marker; lacks sensitivity/specificity

**Table 4:** Preventive measures for CI-AKI.

Cornerstone Measures	Adjunctive/Controversial	Experimental/Emerging
Avoiding volume depletion and nephrotoxic drugs	Oral salt loading	ANP
Use of lowest possible dose of RCM (low-osmolar or nonionic iso-osmolar agents)	NAC	Inorganic nitrate
Fluid administration	Statins	Novel antidiabetic agents
	Diuretics	Oxygenation
	Ascorbic acid	Remote ischemic pre-conditioning
	Vitamin E	Enhanced External Counterpulsation
	Nebivolol	
	Sodium citrate	
	Agents preventing vasoconstrictions	
	Withholding ACE-inhibitors and/or ARB	
	Withdrawal of Metformin	

## Data Availability

No new data were created or analyzed in this study.
